# Aptamer targeted therapy potentiates immune checkpoint blockade in triple-negative breast cancer

**DOI:** 10.1186/s13046-020-01694-9

**Published:** 2020-09-07

**Authors:** Simona Camorani, Margherita Passariello, Lisa Agnello, Silvia Esposito, Francesca Collina, Monica Cantile, Maurizio Di Bonito, Ilya V. Ulasov, Monica Fedele, Antonella Zannetti, Claudia De Lorenzo, Laura Cerchia

**Affiliations:** 1 Institute of Experimental Endocrinology and Oncology “Gaetano Salvatore”, CNR, Via S. Pansini 5, 80131 Naples, Italy; 2 Department of Molecular Medicine and Medical Biotechnology, University of Naples “Federico II”, Via Pansini 5, 80131 Naples, Italy; 3 Ceinge-Biotecnologie Avanzate s.c.a.r.l., via Gaetano Salvatore 486, 80145 Naples, Italy; 4 Pathology Unit, Istituto Nazionale Tumori-IRCCS-Fondazione G. Pascale, Naples, Italy; 5 Group of Experimental Biotherapy and Diagnostic, Institute for Regenerative Medicine, Sechenov First Moscow State Medical University, Moscow, 119991 Russia; 6 Institute of Biostructure and Bioimaging, CNR, Via T. De Amicis 95, 80145 Naples, Italy

**Keywords:** Aptamer, Antitumor immunity, PDGFRβ, PD-L1 monoclonal antibody, TNBC, Tumor microenvironment, Metastases

## Abstract

**Abstract:**

**Background:**

Triple-negative breast cancer (TNBC) is a uniquely aggressive cancer with high rates of relapse due to resistance to chemotherapy. TNBC expresses higher levels of programmed cell death-ligand 1 (PD-L1) compared to other breast cancers, providing the rationale for the recently approved immunotherapy with anti-PD-L1 monoclonal antibodies (mAbs). A huge effort is dedicated to identify actionable biomarkers allowing for combination therapies with immune-checkpoint blockade. Platelet-derived growth factor receptor β (PDGFRβ) is highly expressed in invasive TNBC, both on tumor cells and tumor microenvironment. We recently proved that tumor growth and lung metastases are impaired in mouse models of human TNBC by a high efficacious PDGFRβ aptamer. Hence, we aimed at investigating the effectiveness of a novel combination treatment with the PDGFRβ aptamer and anti-PD-L1 mAbs in TNBC.

**Methods:**

The targeting ability of the anti-human PDGFRβ aptamer toward the murine receptor was verified by streptavidin-biotin assays and confocal microscopy, and its inhibitory function by transwell migration assays. The anti-proliferative effects of the PDGFRβ aptamer/anti-PD-L1 mAbs combination was assessed in human MDA-MB-231 and murine 4 T1 TNBC cells, both grown as monolayer or co-cultured with lymphocytes. Tumor cell lysis and cytokines secretion by lymphocytes were analyzed by LDH quantification and ELISA, respectively. Orthotopic 4 T1 xenografts in syngeneic mice were used for dissecting the effect of aptamer/mAb combination on tumor growth, metastasis and lymphocytes infiltration. Ex vivo analyses through immunohistochemistry, RT-qPCR and immunoblotting were performed.

**Results:**

We show that the PDGFRβ aptamer potentiates the anti-proliferative activity of anti-PD-L1 mAbs on both human and murine TNBC cells, according to its human/mouse cross-reactivity. Further, by binding to activated human and mouse lymphocytes, the aptamer enhances the anti-PD-L1 mAb-induced cytotoxicity of lymphocytes against tumor cells. Importantly, the aptamer heightens the antibody efficacy in inhibiting tumor growth and lung metastases in mice. It acts on both tumor cells, inhibiting Akt and ERK1/2 signaling pathways, and immune populations, increasing intratumoral CD8 + T cells and reducing FOXP3 + Treg cells.

**Conclusion:**

Co-treatment of PDGFRβ aptamer with anti-PD-L1 mAbs is a viable strategy, thus providing for the first time an evidence of the efficacy of PDGFRβ/PD-L1 co-targeting combination therapy in TNBC.

## Background

Triple-negative breast cancer (TNBC), accounting for ~ 15–20% of breast cancers, is a heterogeneous group of tumors with highly metastatic behavior, poor prognosis and an urgent therapeutic need [1, 2]. The lack of expression of oestrogen receptor, progesterone receptor and epidermal growth factor receptor 2 (HER2) makes TNBC difficult to treat leaving chemotherapy as the solely available option for most patients, both in early and advanced stages of the disease, despite its considerable side effects and limited success [3–5]. Nevertheless, thanks to of the continuous effort in searching molecularly targeted approaches for TNBC, the first targeted therapies have been recently approved. These consist in the PARP inhibitors Olaparib or Talazoparib [6], which is beneficial for BRCA-mutated patients but efficiently target TNBC cells regardless of the BRCA-status, and the anti-programmed cell death-ligand 1 (PD-L1) Atezolizumab *plus* nab-paclitaxel chemotherapy [7], which applies to patients with unresectable locally advanced or metastatic PD-L1-positive TNBCs.

The rationale for the latter regimen, representing the first immunotherapy to be approved for the treatment of breast cancer, stands on the significant role of the immune system in TNBC [8]. High PD-L1 expression and amplification of *CD274* (encoding PD-L1) have been found in most TNBC [9] and, along with the presence of tumor-infiltrating lymphocytes (TILs), has been shown to influence TNBC prognosis [10]. Thus, by blocking the interaction of PD-L1, on tumor cells, with PD-1 and B7.1 receptors, on tumor-infiltrating T-cells and antigen-presenting cells, the anti-PD-L1 monoclonal antibody (mAb) Atezolizumab causes a reduction of immunosuppressive signals within the tumor microenvironment (TME). This in turn causes the enhancement of T cell-mediated immunity against tumors [11]. Noteworthy, ongoing clinical studies are exploring combination approaches of various targeting agents together with anti-PD-1/PD-L1 mAbs aimed at maximizing the effectiveness of the treatment, especially for patients with metastatic TNBC, that have only modest response to immune checkpoint inhibitors as monotherapy [12, 13]. Among these, based on preclinical evidence of therapeutic synergy, clinical trials for TNBC treatment are currently underway, or are in recruitment status, by combining anti-PD-1/PD-L1 mAbs with small-molecules inhibitors of receptor tyrosine kinases (RTKs). Some examples are the inhibitors of Axl (NCT03184558), VEGFR (NCT03394287, NCT03797326) and c-Kit (NCT03855358). Furthermore, there is a rapid increase of the number of studies showing the efficacy of co-blocking PD-1, or its ligand, and RTKs in various human cancers [14–17], including TNBC [18].

Platelet-derived growth factor receptor β (PDGFRβ) is a transmembrane RTK expressed on endothelial and perivascular cells, where it plays an important role in wound healing and tissue repair, inflammation and angiogenesis [19]. It is well known that overexpression of PDGFRβ on endothelial cells and tumor-associated stromal cells surface occurs in different human cancers, where the receptor establishes complex signaling pathways inducing angiogenesis and tumor progression [20–22]. Moreover, PDGFRβ expression has been shown as a unique feature of tumor cells characterized by a mesenchymal/stem and poorly differentiated phenotype, and it correlates with aggressiveness and resistance to therapy in multiple tumor types [23–30]. Recent findings prove that PDGFRβ is expressed on the surface of tumor cells belonging to a subgroup of mesenchymal TNBC with invasive and stem-like phenotype and contributes to drive the metastatic potential [31] and vasculogenic mimicry [31, 32] of these tumors.

In searching efficacious strategies to target PDGFRβ-positive TNBC in alternative to PDGFRβ tyrosine kinase inhibitors, which showed limited clinical activity in TNBC as single agents and severe side effects [33, 34], we recently tested the Gint4.T nuclease-resistant RNA aptamer, which we previously validated as a high affinity ligand/inhibitor of PDGFRβ in glioblastoma (GBM) [35, 36] and human bone marrow-derived mesenchymal stem cells (BM-MSCs) [22]. We found that Gint4.T is a potent theranostic agent in TNBC [31], as it efficiently detected lung metastases derived from TNBC cells and suppressed their formation when intravenously administrated in a mouse model [31].

The aim of the present study was to investigate the effectiveness of the combination of the Gint4.T aptamer with anti-PD-L1 antibodies in TNBC since there are no studies including a combined inhibition of both PDGFRβ and PD-1/PD-L1 interaction in the treatment of these tumors. The inhibitory effects of combination of Gint4.T with anti-PD-L1 monoclonal antibodies on tumor cells growth, in monolayer and in co-cultures with lymphocytes, were tested in both human and mouse TNBC cell models. Importantly, we show that the PDGFRβ aptamer augments antitumor immunity and potentiates anti-PD-L1 antibody inhibitory effects on tumor growth and lung metastases formation in 4 T1 TNBC orthotopic mouse model.

## Methods

### Cell cultures

Growth conditions for human breast cancer MDA-MB-231 and BT-474 cell lines, and murine NIH3T3 fibroblasts (American Type Culture Collection, ATCC, Manassas, VA) were previously reported [37]. The murine TNBC 4 T1 cells (ATCC) were grown in Roswell Park Memorial Institute-1640 medium (RPMI-1640, Sigma-Aldrich, Milan, Italy) supplemented with 10% heat-inactivated fetal bovine serum (FBS, Sigma-Aldrich), in 95% air/5% CO2 atmosphere at 37 °C.

Human peripheral blood mononuclear cells (hPBMCs) were isolated and grown as previously described [38, 39]. Mouse lymphocytes were isolated from mouse spleen and grown in R10 medium consisting of RPMI-1640 medium, supplemented with 10% heat-inactivated FBS, 50 U/ml penicillin, 50 μg/ml streptomycin, 2 nM L-glutamine, 10 mM HEPES and 50 mM β-mercaptoethanol.

### Aptamers and monoclonal antibodies

The sequences of the 2’Fluoro-pyrimidines (2’F-Py) RNA PDGFRβ Gint4.T and scrambled (Scr) aptamer, used as negative control, were previously reported [22]. Unlabeled and FAM-labeled aptamers were synthesized by TriLink Biotechnologies (San Diego, CA, USA). 5′-biotinylated Gint4.T and Scr were synthesized by LGC Biosearch Technologies (Risskov Denmark). The handling protocols for aptamers, prior to each treatment, were previously described [31].

Anti-human PD-L1 10_12 mAb, anti-mouse PD-L1 mAb (clone 10F.9G2, BioXcell) and unrelated IgG, used as negative control, were previously reported [40].

### Binding of Gint4.T aptamer to PDGFRβ-positive murine cells

#### Binding affinity (Kd value) calculation

Binding of Gint4.T to 4 T1 cells was assessed by streptavidin-biotin-based assay, as previously described [41]. Briefly, 4 T1 cells (2.0 × 10^4^ cells/well in clear round bottom 96-well plate) were incubated for 10 min at room temperature (RT) with increasing concentrations of 5′-biotinylated Gint4.T or Scr aptamers (10 nM, 20 nM, 50 nM, 100 nM, 200 nM and 500 nM), diluted in the binding buffer (BB) consisting of BlockAid™ blocking solution (Invitrogen, Carlsbad, CA, USA) with 1 mg/ml yeast tRNA and 1 mg/ml ultrapure™ salmon sperm DNA (Invitrogen), as nonspecific competitors. The binding affinity (Kd value) was calculated as previously reported [41], by using Scr to determine the nonspecific binding.

#### Confocal microscopy

To visualize Gint4.T on the surface of PDGFRβ-positive murine cells, 4 T1 and NIH3T3 (1.0 × 10^5^ cells/well in 24-well), previously seeded on a coverslip for 24 h, were incubated with FAM-labeled Gint4.T or FAM-labeled Scr (500 nM-final concentration in BB) for 10 min at RT. PDGFRβ-negative BT-474 cells were treated in the same condition and used as negative control. After three washes in Dulbecco’s phosphate-buffered saline (DPBS), cells were fixed with 4% paraformaldehyde in DPBS for 20 min, washed three times in DPBS and incubated with 1.5 μM 4′,6-Diamidino- 2-phenylindole (DAPI, D9542, Sigma-Aldrich). Finally, coverslips were mounted with glycerol/DPBS. The fluorescence images were taken under a Zeiss LSM 700 META confocal microscopy equipped with a Plan-Apochromat 63x/1.4 Oil DIC objective.

### Inhibition of murine cells migration by Gint4.T aptamer

4 T1 and NIH3T3 cells were serum starved overnight in the presence of 500 nM Gint4.T or Scr. Then, cells (4 T1, 5 × 10^4^/well; NIH3T3, 2 × 10^5^/well) were seeded into the upper chamber of a 24-well transwell (Transwell filters 8 μm pore size; Corning Incorporate, Corning, NY) in the presence of 500 nM Gint4.T or Scr and exposed to medium containing PDGF-BB (50 ng/ml, R&D Systems, Minneapolis, MN), as inducer of migration. After incubation for 24 h at 37 °C in a humidified incubator in 5% CO2, the migrated cells were visualized by staining with 0.1% crystal violet in 25% methanol and photographed. Stained cells were lysed in 1% sodium dodecyl sulfate and absorbance at 595 nm was measured on a microplate reader.

### PDGF-BB stimulation of murine cells

NIH3T3 cells (1.5 × 10^5^ cells/well in 6-well) were mock-treated or serum-starved for 18 h and then left untreated or stimulated for 10 min with 20 ng/mL PDGF-BB. Cell lysates preparation and immunoblotting analyses with anti-phospho-PDGFRβ (Tyr771, indicated as p-PDGFRβ) (Cell Signaling Technology Inc.) primary antibodies were performed as previously reported [42] by using anti-vinculin (N-19) (Santa Cruz Biotechnology, Santa Cruz, CA) as loading control. Blots shown are representative of at least three independent experiments.

### Cell growth inhibition by Gint4.T aptamer/anti-PDL1 mAb combined treatment

MDA-MB-231 (5.0 × 10^3^ cells/well) and 4 T1 (3.0 × 10^3^ cells/well) were plated in 96-well and, after 16 h at 37 °C, were either untreated or treated for 96 h with 200 nM Gint4.T and 100 nM human anti-PD-L1 10_12 (MDA-MB-231) or murine anti-mPD-L1 (4T1), used alone or in combination. Unrelated IgG (100 nM) and Scr (200 nM) were used as negative controls. The treatment with the aptamers was renewed after 72 h. Cell counts were measured by the trypan blue exclusion test.

### Effects of combinatorial treatments on co-cultures of tumor cells and lymphocytes

MDA-MB-231 (1.5 × 10^4^ cells/well) or 4 T1 (1.5 × 10^4^ cells/well) cells, previously seeded in 96-well flat-bottom plates for 16 h at 37 °C, were co-cultured with human or murine lymphocytes, respectively, at effector:target cells ratio 10:1, in the absence or presence of 200 nM Gint4.T or 100 nM anti-PD-L1 10_12 (human setting) or anti-mPD-L1 (mouse setting), used alone or in combination. Unrelated IgG (100 nM) and Scr (200 nM) were used as negative controls. After 24 h incubation at 37 °C in a humidified incubator in 5% CO2, lymphocytes were removed and adherent cells were washed and counted by the trypan blue exclusion test.

For determination of tumor cell lysis, the release of lactate dehydrogenase (LDH) in the cellular co-culture supernatants was measured by a LDH detection kit (Thermofisher Scientific, Meridian Rd., Rockoford, IL, USA), as previously described [38, 39].

The concentration of interleukin-2 (IL-2) or interferon gamma (IFN-γ) cytokines secreted in the cellular co-cultures supernatant was measured by ELISA assays (DuoSet ELISA, R&D Systems, Minneapolis, MN, USA), as previously described [38, 39].

### Binding of Gint4.T aptamer to human and mouse lymphocytes

Binding of 5′-biotinylated Gint4.T to human or mouse activated lymphocytes was assessed as previously described [38] by using increasing concentrations (50 nM, 100 nM and 200 nM) of 5′-biotinylated Gint4.T or Scr aptamers.

### In vivo experiments

4 T1 cells (3 × 10^4^) were re-suspended in 0.1 ml of 1:1 mix of physiological saline and Matrigel (BD Biosciences, Franklin Lakes, NJ) and orthotopically injected into the mammary fat pads of five-week-old Female Balb/c mice, which weighed about 20–22 g (Charles River, Milan, Italy). Once tumors became approximately 150 mm^3^ [volume = 0.5 × long diameter × (short diameter)^2^], mice were randomized into four groups (five mice for each group): Ctrl (1400 pmol Scr/intravenous injection, at day 0, 2, 4, 7 and 9); Gint4.T aptamer (1400 pmol Gint4.T/intravenous injection, at day 0, 2, 4, 7 and 9); anti-mPD-L1 (200 μg/intraperitoneal injection, at day 0, 4 and 9); Gint4.T *plus* anti-mPD-L1 (1400 pmol Gint4.T/intravenous injection at day 0, 2, 4, 7 and 9 *plus* 200 μg anti-mPD-L1/intraperitoneal injection, at day 0, 4 and 9). The long and short diameters of the tumors were measured using slide calipers up to day 11 (2 days after the last treatment) and the body weight was also measured. At day 11, mice were euthanized. Treatment schedule is schematized in Fig. 4a.

### Ex vivo analyses

After sacrificing mice, tumors from each animal were excised and cut into two pieces for sample processing: one piece was stored in 10% neutral buffered formalin for immunohistochemistry analyses, and the other piece was frozen, using liquid nitrogen, for RNA extraction and reverse transcription quantitative polymerase chain reaction (RT-qPCR) and protein lysis for immunoblotting analyses. Lung from each animal were harvested and stored in 10% neutral buffered formalin for immunohistochemistry analyses.

#### RT-qPCR

RNA extraction and RT-qPCR were performed as previously described [31] on tumors from four animals per group. Primers used were: IL-2, Fwd 5′-TTGTCGTCCTTGTCAACAGC-3′, Rev. 5′- CTGGGGAGTTTCAGGTTCCT-3′; IFN-γ, Fwd, 5′-AGCGGCTGACTGAACTCAGATTGTAG-3′, Rev. 5′-GTCACAGTTTTCAGCTGTATAGGG-3′. The following primers were used for internal control: Glucose-6-phosphate dehydrogenase, Fwd 5′-TTATCATCATGGGTGCATCG-3′, Rev. 5′-GCATAGCCCACAATGAAGGT-3′.

#### Immunoblotting analyses on tumor lysates

Tumor lysates preparation and immunoblotting analyses were performed as previously reported [42]. Filters were probed with the indicated primary antibodies: anti-PDGFRβ, anti-phospho-44/42 MAPK (extracellular signal-regulated kinase 1/2, ERK1/2, indicated as p-ERK1/2), anti-phospho-Akt (Ser473, indicated as p-Akt), anti-Akt (Cell Signaling Technology Inc.), anti-PD-L1/CD274 (Proteintech Group, Inc.), anti-ERK1 (C-16) and anti-vinculin (N-19) (Santa Cruz Biotechnology, Santa Cruz, CA). Blots shown are representative of at least three independent experiments.

#### Immunohistochemistry

Formalin-fixed tumors and lungs were paraffin-embedded and sectioned (4 μm) and three samples per group were stained with hematoxylin and eosin (H&E) or immunostained as reported [31]. The primary antibodies used were: anti-CD8 alpha antibody (clone D4W2Z, #98941S, dilution 1:500, 1 h 4 °C incubation, Cell Signaling Technology Inc.); anti-FoxP3 (clone D608R, #12653S, dilution 1:150, 1 h 4 °C incubation, Cell Signaling); anti-Granzyme B (GRZB ab4059; dilution 1:150 diluted, 4 °C RT, Abcam, Cambridge, MA); anti-Ki-67 (dilution 1:75, overnight 4 °C incubation, Invitrogen); anti-PDGFRβ (diluition 1:50, 1 h 4 °C incubation, Cell signaling Technology Inc.); anti-PD-L1 (diluition 1:50, 1 h 4 °C incubation, Proteintech Group).

Results were interpreted using Olympus BX43 light microscope (Olympus, Center Valley, PA). Each slide was reviewed blinded and, to ensure accuracy, the number of metastases and of Ki-67, CD8, GRZB and Foxp3-positive cells was determined by two independent counts.

### Statistical analysis

Statistical values were defined using GraphPad Prism version 8.00 by unpaired *t-test* (two variables) or one-way ANOVA followed by Tukey’s multiple comparison test (more than two variables). *P* value < 0.05 was considered significant for all analyses.

## Results

### The PDGFRβ aptamer enhances the anti-proliferative activity of anti-PD-L1 mAb on human TNBC cells

As both PDGFRβ and PD-L1 are crucial targets for TNBC treatments [31, 43, 44], we tested whether their co-inhibition would enhance TNBC cells killing. To this aim, we used an aptamer and a mAb that we previously validated as high affinity binders and inhibitors of PDGFRβ [31] and PD-L1 [40, 45], respectively, in different tumor types including TNBC. Specifically, Gint4.T is a 2’F-Py RNA aptamer that binds to the extracellular domain of PDGFRβ and inhibits receptor activation and downstream phosphatidyl-3-kinase (PI3K)/Akt and ERK1/2 pathways [22, 31, 35], thus affecting growth, migration and invasion of mesenchymal TNBC cell lines [31]. On the other side, the high affinity human anti-PD-L1 (10_12) mAb [38, 46] not only interferes in the PD-L1/PD-1 interaction but also inhibits the immune-independent PD-L1 positive TNBC cell growth by affecting the intracellular MAPK pathway [40].

**Fig. 1:**
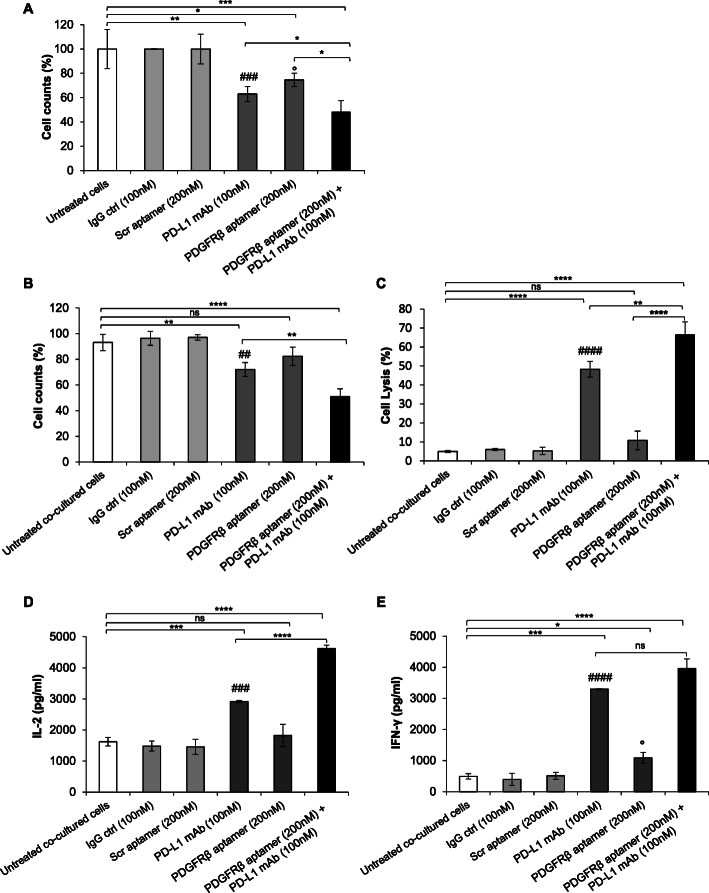
Effects of combinatorial treatments on human MDA-MB-231 cells in the absence or presence of lymphocytes. **a** Cell growth inhibition of MDA-MB-231 cells, untreated or treated with the anti-PD-L1 (10_12) mAb or the PDGFRβ aptamer, used alone (dark grey bars) or in combination (black bars), for 96 h at 37 °C at the indicated concentrations. Untreated cells (white bars) or cells treated with an unrelated IgG or Scr (light grey bars) were used as negative controls. Data were expressed as percentage of viable treated cells with respect to untreated cells. **b-e** MDA-MB-231 cells were co-cultured with human lymphocytes and left untreated or treated for 24 h, as indicated. **b** Cell counts were expressed as percentage of viable treated cells with respect to untreated co-cultured cells. **c** Cell lysis was expressed as measure of LDH release and reported as percentage of lysis of treated cells with respect to the untreated cells, used as a control. **d** IL-2 and **e** IFN-γ secretion levels were measured by ELISA on supernatants of cells treated, as indicated. **a-e** Error bars depict mean ± SD (*n* = 3). ^####^*P* < 0.0001, ^###^ *P* < 0.001, ^##^ *P* < 0.01 relative to IgG; °*P* < 0.05 relative to Scr; ^****^P < 0.0001, ^***^ *P* < 0.001, ^**^ *P* < 0.01, ^*^P < 0.05; one-way ANOVA followed by Tukey’s multiple comparison test

First, we analyzed the effects of Gint4.T aptamer and 10_12 mAb used in combination, along with single agents, on the growth of human TNBC MDA-MB-231 cells, expressing high levels of both PDGFRβ [31, 37] and PD-L1 [40], further enhanced by previous cell exposure to cisplatin and doxorubicin, two chemotherapeutics used in clinic for TNBC [41]. Based on our previous dose and time-response analyses in MDA-MB-231 cells for the aptamer [31] and antibody [40], we used them at a concentration of 200 and 100 nM, respectively, prolonging the incubation up to 96 h. Indeed, under these experimental conditions the inhibitory effects of both the aptamer and antibody is not so strong as it is at higher concentration and/or longer incubation time [31, 40], thus allowing to appreciate a potential enhancement of their effects in combination. As shown in Fig. 1a, the combined treatment led to a significantly stronger reduction of cell growth (50% inhibition), compared to either the single anti-PD-L1 antibody (37% inhibition) or anti-PDGFRβ aptamer (25% inhibition). As expected, no effects were observed in the presence of an unrelated IgG antibody and a scrambled aptamer (Scr), used as negative controls. Next, in order to assess whether the aptamer-mediated inhibition of PDGFRβ enhances the inhibitory effects on cell growth exerted by 10_12 mAb in the context of PD-1/PD-L1 interaction between TNBC cells and infiltrating lymphocytes [38, 40], we tested the aptamer *plus* mAb on co-cultures of MDA-MB-231 cells with human lymphocytes by using hPBMCs (effector:target ratio 10:1) (Fig. 1b-e). Noteworthy, following 24 h-treatment, Gint4.T even though not exerting itself an appreciable reduction of cell growth when used as single agent, it caused a significant increase of the 10_12 mAb inhibitory effect on cell growth, that reached about 50% inhibition with respect to 28% inhibition observed when the mAb was used alone (Fig. 1b). Further, the co-treatment of the cells with Gint4.T aptamer and 10_12 mAb had a more potent cytotoxic effect, as assessed by higher levels of LDH released by tumor cells co-cultured with lymphocytes, than that observed with the sole anti-PD-L1 antibody (Fig. 1c). Moreover, a significant increase of IL-2 (Fig. 1d) and IFN-γ (Fig. 1e) secretion was detected when MDA-MB-231 cells were co-cultured with lymphocytes and treated with 10_12 with respect to untreated co-cultured cells, with IL-2 secretion that was further increased by the combined Gint4.T/10_12 treatment (Fig. 1d). Accordingly to previous findings showing that PDGFRβ is expressed on T cells and PDGF-BB, its primary ligand, potently regulates T cells function [47], we found that Gint4.T binds to the receptor on the surface of activated lymphocytes, as measured by cell-ELISA assays (Figure S1A), thus suggesting that it may modulate cytokines secretion by lymphocytes.

### The PDGFRβ aptamer binds to and inhibits the murine receptor

**Fig. 2:**
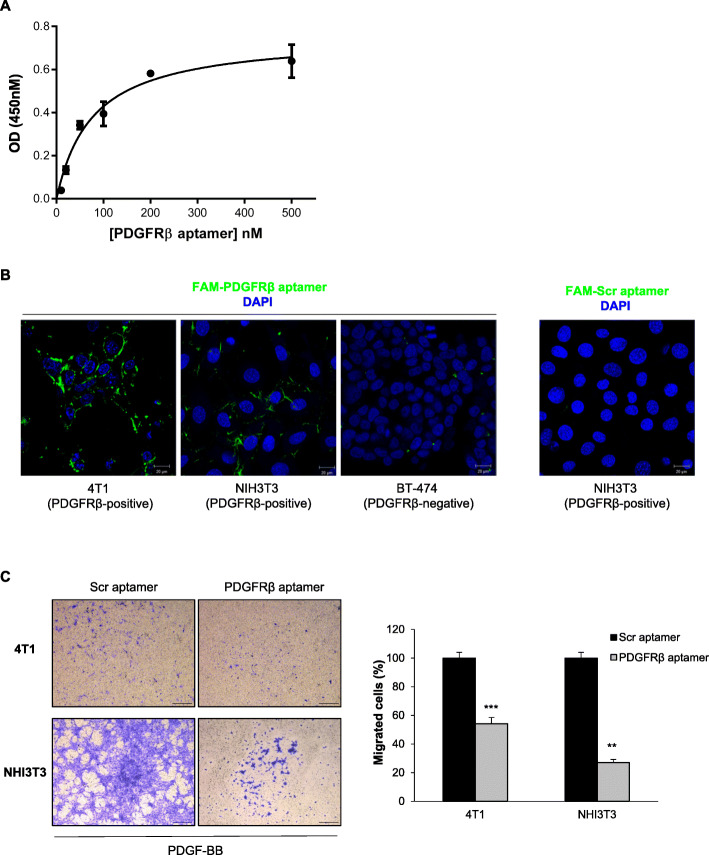
Gint4.T aptamer specifically binds to and inhibits migration of PDGFRβ-positive murine cells. **a** Binding curve of 5′-biotinylated PDGFRβ Gint4.T aptamer to 4 T1 cells for calculation of the apparent Kd of aptamer-cell interaction. The background binding value for 5′-biotinylated Scr sequence was subtracted from each data point. Data shown are mean ± SD (*n* = 3). **b** Representative confocal images of 4 T1, NIH3T3 and BT-474 cells incubated with FAM-Gint4.T or FAM-Scr, as indicated. FAM-aptamers and nuclei are visualized in green and blue, respectively. All digital images were captured at the same setting to allow for direct comparison of staining patterns. Magnification 63 ×, scale bar = 20 μm. **c** *Left*, 4 T1 and NIH3T3 cell migration toward PDGF-BB was analyzed by transwell migration assay in the presence of Gint4.T or Scr. Photographs of a representative experiment are shown. Magnification 5 ×, scale bar = 500 μm. *Right*, data were presented as percentage of migrated cells in the presence of Gint4.T with respect to cells treated with Scr. Bars depict mean ± SD (*n* = 3). ^***^ *P* < 0.001, ^**^ *P* < 0.01 relative to Scr; unpaired *t-test*

In order to test the promising aptamer/mAb combination treatment in vivo, by exploiting a fully immunocompetent murine preclinical syngeneic model, first we checked the capability of Gint4.T aptamer, selected to recognize the human receptor [35], to bind to PDGFRβ-positive 4 T1 cells, an established mouse model for aggressive TNBC cells [48, 49]. Even if some species specificity has been shown in aptamers recognition ability [50], we hypothesized a high affinity binding for Gint4.T aptamer to murine PDGFRβ because it shares about 80% sequence identity in the extracellular region with the human receptor (Figure S2A) and, accordingly, the recombinant human PDGF-BB ligand, efficiently stimulates the murine receptor expressed on PDGFRβ-positive NIH3T3 cells (Figure S2B). As expected, Gint4.T efficiently binds to 4 T1 cells with a Kd value of 76.76 ± 14.69 nM (Fig. 2a), which was comparable to that observed on human MDA-MB-231 cells (57.48 ± 7.8 nM) [31]. Confocal microscopy analyses on both 4 T1 and NIH3T3 cells confirmed the ability of FAM-labeled Gint4.T to decorate the surface of murine cells (Fig. 2b). Incubation of Gint4.T on PDGFRβ-negative BT-4 T4 breast cancer cells [31] and of Scr on NIH3T3 cells (Fig. 2b) were used as negative controls in parallel analyses.

Next, given the driving role of PDGFRβ on cell motility and the ability of Gint4.T to hamper migration and invasion of human cancer cells [22, 31, 35], to confirm its effect on murine receptor upon binding, we proved that the aptamer strongly reduces the PDGF-BB-stimulated migration of 4 T1 and NIH3T3 cells, reaching about 45 and 70% inhibition, respectively, compared to the Scr aptamer (Fig. 2c).

Overall, these results, showing mouse-human cross-reactivity of Gint4.T, make the aptamer a valuable tool for preclinical therapeutic and/or diagnostic evaluation studies in murine environments.

### Efficacy of PDGFRβ and PD-L1 co-blockade on murine TNBC cells

**Fig. 3:**
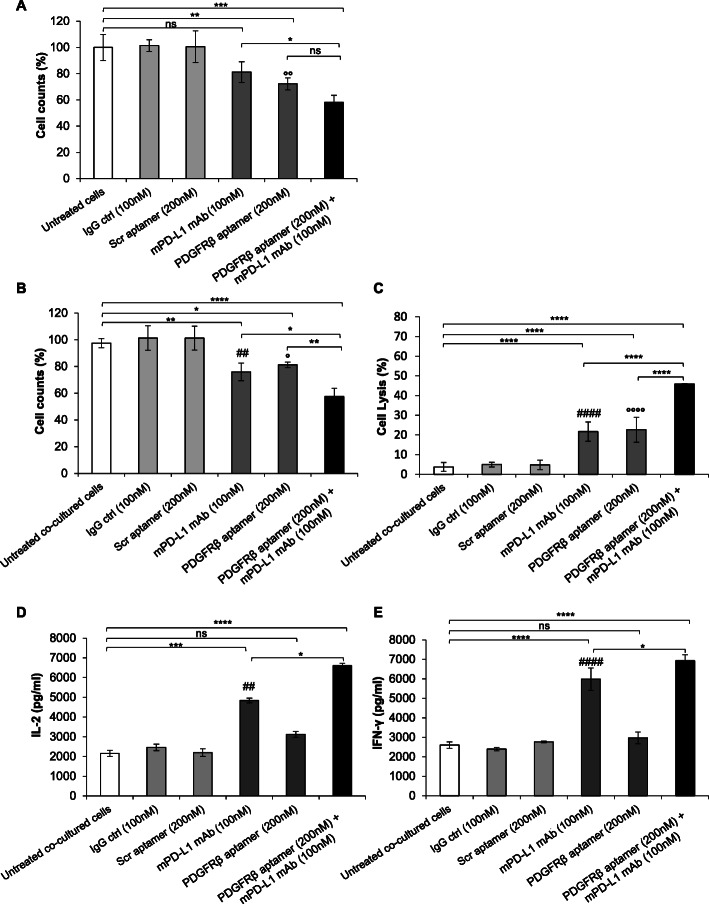
Effects of combinatorial treatments on murine 4 T1 cells in the absence or presence of lymphocytes. **a** Cell growth inhibition of 4 T1 cells, untreated or treated with the anti-mPD-L1 mAb or the PDGFRβ aptamer, used alone (dark grey bars) or in combination (black bars), for 96 h at 37 °C at the indicated concentrations. Untreated cells (white bars) or cells treated with an unrelated IgG or Scr (light grey bars) were used as negative controls. Data were expressed as percentage of viable treated cells with respect to untreated cells. **b-e** 4 T1 cells were co-cultured with mouse lymphocytes and treated for 24 h, as indicated. **b** Cell counts were expressed as percentage of viable treated cells with respect to untreated co-cultured cells. **c** Cell lysis was measured by LDH release in the medium after incubation with the indicated compounds. **d** IL-2 and **e** IFN-γ secretion levels were measured by ELISA on supernatants of cells treated as indicated. **a-e** Error bars depict mean ± SD (*n* = 3). ^####^ *P* < 0.0001, ^##^ *P* < 0.01 relative to IgG; ^°°°°^ *P* < 0.0001, ^°°^ *P* < 0.01, ° *P* < 0.05 relative to Scr; ^****^ *P* < 0.0001, ^***^ *P* < 0.001, ^**^ *P* < 0.01, ^*^ *P* < 0.05; one-way ANOVA followed by Tukey’s multiple comparison test

Before moving to the in vivo experiments, we first verified the effectiveness of PDGFRβ and PD-L1 combined inhibition on murine 4 T1 cells in vitro. Considering the low cross-reactivity of 10_12 mAb for the murine PD-L1 [40], we used a validated anti-mouse PD-L1 mAb (clone 10F.9G2, BioXcell) for PD-L1 blocking [51]. Under the same treatment conditions as for MDA-MB-231 cells, which gave rise to just a slight and not even significant inhibition of 4 T1 cell growth in monolayer by anti-mPD-L1, the combination of Gint4.T and anti-mPD-L1 had a greater effect, which was significant compared to the single antibody treatment while there was a trend toward a greater beneficial compared with the aptamer alone that had already a significant effect as a single agent (Fig. 3a). According to the results obtained with human cells, the potentiated effects of the combinatorial approach over the use of single agents were also observed in co-cultures of tumor cells with mouse lymphocytes on both cell growth (Fig. 3b) and lysis (LDH release) (Fig. 3c). Finally, the co-treatment of the cells with Gint4.T and anti-mPD-L1 mAb resulted in an increased secretion of both IL-2 and IFN-γ compared with either compound alone (Fig. 3d). Interestingly, as previously observed on the human cells, Gint4.T binds to activated mouse lymphocytes (Figure S1B) which is consistent with the observed cytotoxic effects (Fig. 3b and c) caused by the aptamer in co-cultures experiments when used as single agent, and with its ability to potentiate the antibody effect in the combined treatment (Fig. 3d and e).

### Targeting PDGFRβ in vivo potentiates anti-PD-L1 efficacy modifying the tumor microenvironment

Next, in vivo therapeutic effects of the combinatorial anti-PDGFRβ and anti-PD-L1 treatment was evaluated by using orthotopic 4 T1 xenografts in syngeneic BALB/c model, representing the TNBC subtype [48, 52, 53].

**Fig. 4:**
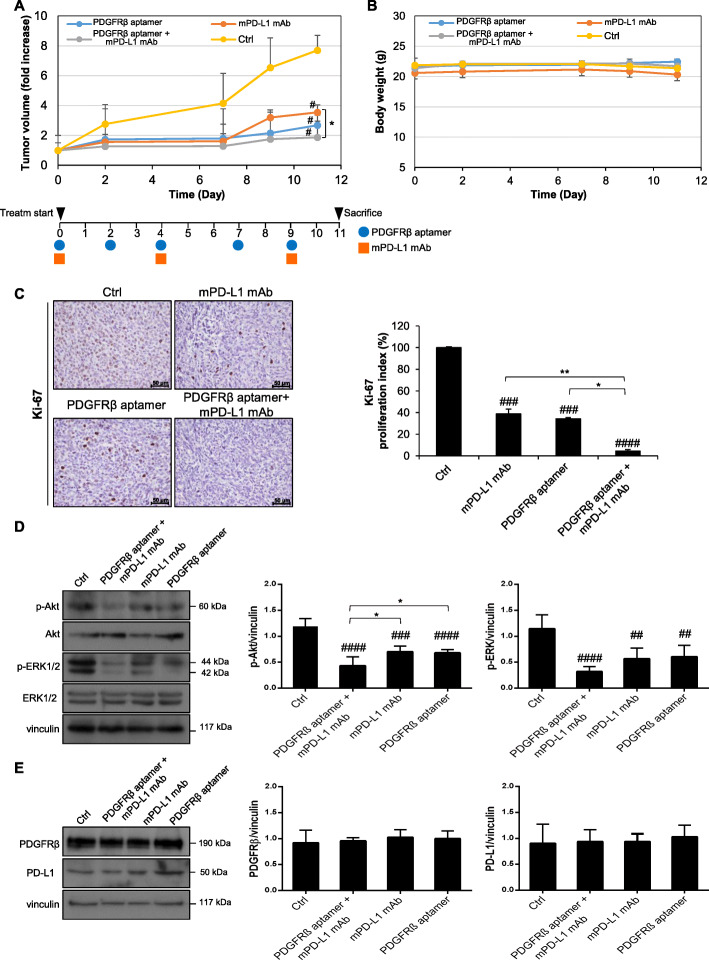
Effects of combinatorial treatments on tumor growth. **a** Mice bearing 4 T1 orthotopic xenografts were injected intravenously with PDGFRβ Gint4.T aptamer (at day 0, 2, 4, 7 and 9) and intraperitoneally with anti-mPD-L1 mAb (at day 0, 4 and 9), alone and in combination. Mice treated with Scr aptamer were used as the control group (Ctrl). Tumor growth was monitored by calipers over time and experimental raw data (expressed as fold increase) were interpolated with no curve fitting or regression analysis. Treatment schedule is shown. Day 0 marks the start of treatments. ^#^ *P* < 0.05 relative to Ctrl; one-way ANOVA followed by Tukey’s multiple comparison test; ^*^ *P* < 0.05. **b** Mice body weight was measured at the indicated days and the mean weight of each group is shown. **a**-**b** The mean ± SEM (*n* = 5) were calculated for all the groups. **c** Shown are images from one representative tumor sample for each treatment group stained with Ki-67 antibody. Ki-67 proliferation index was calculated as percentage of Ki-67 positive cells/total cell count for 5 randomly selected 40 × microscopic fields considering the Ctrl-group as 100%. Magnification 40 ×, scale bar = 50 μm. **d**-**e** *Left*, lysates from recovered tumors were immunoblotted with the indicated antibodies. Equal loading was confirmed by immunoblot with anti-vinculin antibody. One representative tumor sample per group is shown. Molecular weights of indicated proteins are reported. *Middle and Right*, quantification of immunoblot analysis for p-Akt, p-ERK1/2, PDGFRβ and PD-L1 normalized to the loading control vinculin. Bars depict mean ± SD (five mice for each group). **c**-**e** ^####^ *P* < 0.0001, ^###^ *P* < 0.001, ^##^ *P* < 0.01 relative to Ctrl;^**^ *P* < 0.01; ^*^ *P* < 0.05; one-way ANOVA followed by Tukey’s multiple comparison test

Treatments were initiated at 14 days after cell inoculation, when tumor mean volume was ~ 150 mm^3^, and tumor growth was monitored by a caliper measuring tumor size for further 12 days. The anti-PDGFRβ aptamer (at a dose of 1400 pmol, 0.74 mg/kg mean body-weight, at day 0, 2, 4, 7 and 9) and anti-mPD-L1 mAb (at a dose of 10 mg/kg mean body-weight, at day 0, 4 and 9) were administered intravenously or intraperitoneally, respectively, alone and in combination, according to the reference dosage of the aptamer [31] and antibody [40, 51], as single agents, for mice treatment and that of Atezolizumab and other immunomodulators in clinic [54]. Mice treated with scrambled aptamer served as control group (Ctrl). The results showed a significant inhibitory effect on tumor growth in mice treated with either Gint4.T or anti-mPD-L1, both if administrated alone or in combination, with respect to control group, which formed faster-growing tumors (Fig. 4a). Interestingly, the aptamer significantly enhanced the efficacy of the antibody in the combined treatments (1.87 ± 1.08 vs 3.54 ± 0.66, Gint4.T *plus* anti-mPD-L1 vs anti-mPD-L1, *P* = 0.016). The treatments were well tolerated in vivo, with no significant loss in mice body weight over the entire treatment period (Fig. 4b). Also, the tumor proliferative potential was drastically hampered by Gint4.T *plus* anti-mPD-L1 treatment compared with either single agent as assessed by immunohistochemical staining for Ki-67-positive cells in the tumors (Fig. 4c). Next, accordingly to the strong inhibition of tumor growth, immunoblotting analyses performed on lysates from treated tumors showed a marked inhibition of Akt and ERK1/2 pathways, downstream of both PDGFRβ [22, 31, 35] and oncogenic PD-L1 [40, 55], in the tumors from mice treated with the aptamer, antibody and their combination (Fig. 4d). Interestingly, the combined treatment decreased the extent of Akt and ERK1/2 phosphorylation more efficiently than the treatment with aptamer or anti-PD-L1 mAb alone, showing a significant or a decreasing trend, respectively (Fig. 4d). No changes of PDGFRβ and PD-L1 expression were observed upon treatments as assessed both by immunoblotting (Fig. 4e) and immunohistochemistry (Figure S3).

**Fig. 5:**
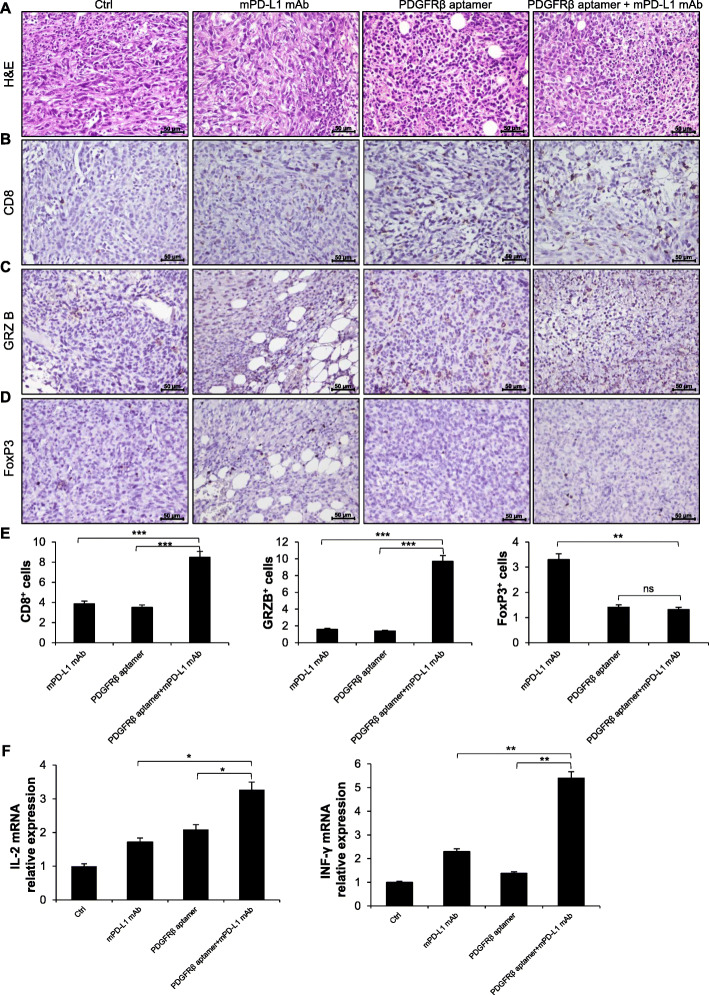
Effects of combinatorial treatments on tumor-infiltrating lymphocytes. Shown are images from one representative tumor sample for each treatment group stained for H&E (**a**) and immunostained with anti-CD8 (**b**), anti-GRZB (**c**) and anti-FoxP3 (**d**) antibodies. Magnification 40 ×, scale bar = 50 μm. **e** CD8+, GRZB+ and FoxP3+ cell counts in the tumors of anti-mPD-L1, PDGFRβ aptamer and PDGFRβ aptamer *plus* anti-mPD-L1 groups expressed relative to cell counts in Ctrl-tumors. **f** RNA extracted from recovered tumors was analyzed by RT-qPCR for the IL-2 and INF-γ genes and mRNA relative expression was reported. Bars depict mean ± SD (*n* = 4). **e**-**f** ^***^ *P* < 0.001; ^**^ *P* < 0.01, ^*^ *P* < 0.05; one-way ANOVA followed by Tukey’s multiple comparison test

Next, immunohistochemical analyses were performed on tumors from treated groups and compared with those from the Ctrl group. First, H&E staining revealed the high extent of lymphocytes infiltration in the tumors, which is expected for high aggressive TNBC (Fig. 5a). Then, we stained tumors with antibodies specific for anti-CD8 and FoxP3-expressing regulatory T (Treg) cells, and GRZB, established indicators of antitumor activity [56]. As shown, we found an increase of both intratumoral CD8+ T cells (Fig. 5b and e) and GRZB positive staining (Fig. 5c and e) following Gint4.T *plus* anti-mPD-L1-treatment with respect to anti-PD-L1 or PDGFRβ aptamer single treatments. Conversely, a reduction in the number of Treg cells, as assessed by quantification staining of FoxP3, was observed when using either PDGFRβ Gint4.T aptamer alone or combining the Gint4.T treatment with the anti-PD-L1 compared to anti-PD-L1 alone (Fig. 5e). This is consistent with the findings that depletion of Treg cells is effective in evoking antitumor immune responses by promoting tumor infiltration of CD8+ T cells with tumor-specific killing activity [57]. Further, consistent with the findings obtained in vitro (Fig. 3d and e), the combined treatment of the aptamer and antibody significantly increased the levels of both IL-2 and IFN-γ compared with each single treatment, as assessed by RT-qPCR on tumor samples (Fig. 5f).

**Fig. 6:**
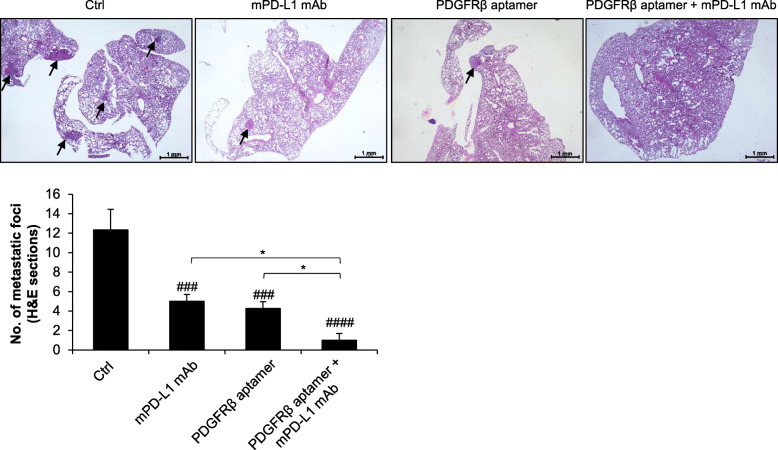
Effects of combinatorial treatments on lung metastases formation. Shown are images from one representative lung sample for each treatment group stained for H&E (Magnification 2 ×). Metastatic foci (indicated by the arrows) in H&E lung sections were counted under a light microscope (Magnification 10 ×). Bars depict mean ± SD. ^####^ *P* < 0.0001, ^###^ *P* < 0.001 relative to Ctrl; * *P* < 0.05; one-way ANOVA followed by Tukey’s multiple comparison test

The 4 T1 tumor is highly metastatic, with lungs as the principal target organ [48, 53, 58], and depletion of CD8+ T cells induces metastases formation in this model [52]. Notably, large metastatic foci were present in the lungs from the Ctrl group, whereas they were significantly reduced in those treated with Gint4.T or anti-mPD-L1 mAb and almost undetectable in the lungs of aptamer/mAb-treated mice (Fig. 6).

## Discussion

Although immunotherapy approaches based on immune checkpoint inhibitors such as anti-PD-1 or anti-PD-L1 antibodies have shown convincing results in multiple cancers, they are active in only a minority of patients [59]. Thus, multiple combination approaches currently aim to improve the efficacy of PD-1/PD-L1 blockade counteracting the immunosuppressive effect of the TME and avoiding the occurrence of therapeutic resistance. However, this often requires the use of cytotoxic chemotherapy, as in the case of metastatic TNBC, which may cause negative systemic side effects. In order to address this shortcoming, the ideal anti-cancer treatment alongside immunotherapy should include tumor-specific targeting agents that exhibit significant therapeutic effects and high tissue penetration. In that regards, one of the latest trends in oncotherapy is the use of aptamers, representing one of the most promising compounds able to specifically target tumor markers [60–62]. They are single-stranded oligonucleotides that, resembling antibodies, utilize their tridimensional shape for target recognition [63, 64]. Active tumor targeting by aptamers, while preserving affinity and specificity similar to mAbs, presents several advantages over them, including smaller size, higher stability, cheaper cost for synthesis, minimal inter-batch variability and lack of immunogenicity [61, 65–67]. While aptamers are generally functionalized with cytotoxic payloads for cancer therapy, it has been also demonstrated their ability to be antagonistic agents independent of drug conjugation, exerting significant potential as anti-cancer therapeutics [31, 37, 68]. PDGFRβ has been shown as an ideal target for antagonistic therapy development in aggressive human cancers, including TNBC [23–31]. Herein, the PDGFRβ antagonist Gint4.T aptamer [22, 31, 35] strengthens anti-PD-L1 antibody therapeutic efficacy in both human and murine TNBC cell cultures and in a well-established mouse model for TNBC. Our results clearly show that there are multiple mechanisms through which PDGFRβ aptamer could potentiate anti-PD-L1 antibody effects. It indeed causes inhibition of tumor growth and metastases formation by a direct effect on tumor cells, and also augments tumor immunity, which might secondarily facilitate the anti-tumor activity of anti-PD-L1 antibodies. The mechanism of action of the Gint4.T aptamer as both high specific targeting agent of human PDGFRβ expressed on the surface of tumor cells and inhibitor of receptor activation and downstream dependent ERK1/2 and PI3K/Akt signaling pathways in GBM [35, 36, 42] and TNBC [31], has been previously clarified and reported in literature and here confirmed for the first time in murine cells. Indeed, we show that Gint4.T is able to bind to murine PDGFRβ-positive cells thus hampering cell growth and migration. Accordingly, a strong reduction of tumor growth and lung metastases was observed in orthotopic 4 T1 mouse xenografts in syngeneic BALB/c mice, which was accompanied by a strong inhibition of both ERK1/2 and Akt signaling molecules in tumor samples. Also, we suggest that Gint4.T acts on immune populations causing both the depletion of Treg cells and the increase of CD8^+^ T cells tumor infiltration together with an increase of GRZB, thus heightening the antibody-dependent antitumor immunity. Consistently, in both in vitro human and murine co-cultures of TNBC cells and lymphocytes and in vivo 4 T1 xenograft model, the aptamer potentiates the anti-PD-L1 antibody-induced T cell stimulation [46], as demonstrated by the increase in the levels of IL-2 and IFN-γ cytokines. Accordingly, it has been shown that PDGF may act directly on certain lymphocyte subsets [47, 69, 70]. Further, the increase of Tregs in breast tumors correlates with an invasive phenotype and a poor prognosis, and aggressive TNBC is considerably associated with high expression of Tregs [71]. Notably, small molecules inhibitors of PDGFRβ have been reported to augment tumor immunity by depletion of FoxP3-expressing Tregs and increase in CD8^+^ T cells in humans and advanced tumor-bearing mice [72–74].

Tumor development and progression are promoted by the establishment of a favorable TME, including PDGFRβ-positive mesenchymal stem cells (MSCs), tumor-associated fibroblasts, angiogenic endothelial cells, and infiltrating immune cells, through a cytokine network [75–77]. It is likely that, by acting on these cell components, Gint4.T modifies TME to ultimately potentiate the anti-PD-L1 responses. At this regard, we previously showed that Gint4.T aptamer binds to and inhibits PDGFRβ on BM-MSCs, thus hampering their homing into TNBC TME and consequently counteracting bone marrow-derived MSCs role to enhance lung metastases formation [22]. Also, we showed that the aptamer is able to transcytose the blood-brain barrier (BBB), by binding to PDGFRβ highly expressed on endothelial cells of vessels that vascularize the tumor [36]. We could thus speculate that the aptamer may interfere with tumor vessels formation thereby inhibiting tumor growth and metastatic potential. Furthermore, because the preferential expression of PDGFRβ by cancer cells with stem-like characteristics and/or that have undergone epithelial-mesenchymal transition (EMT) [31, 61], it would be interesting to assess whether the proposed aptamer-based strategy results in reducing the proportion of mesenchymal, stem-like cells. In that case it would also counteract the recently evidenced detrimental effects of chemotherapy on tumor relapse and metastasis promotion due to induction of EMT and stemness phenotype [78].

These findings provide rationale for the combined therapeutic targeting of PDGFRβ and PD-L1 in TNBC, where immune-checkpoint blockade as single therapy has met limited success so far.

Further, they lay the ground to construct a new bispecific immunoconjugate, made up of anti-PD-L1 antibody covalently linked to Gint4.T aptamer, thus optimizing the efficacy of the combination therapy by increasing their co-targeting at the tumor site while dispensing lower doses of either single agents and overcoming the limits related to the rapid clearance of the aptamers. We previously developed three different bispecific conjugates consisting of EGFR aptamer linked to either anti-HER2, anti-PD-L1 or anti-CTLA-4 mAbs [38, 39]. By this strategy the advantages of Gint4.T aptamer (nuclease resistance, rapid tumor uptake, durable tumor retention [31] and anti-PD-L1 antibodies (longer half-life in circulation, immunomodulatory activity) could be combined in one single molecule with improved therapeutic effectiveness and pharmacokinetic/pharmacodynamic properties over the parental moieties. At this regard, it would be intriguing to assess whether the conjugation of the PDGFRβ aptamer with a fragment smaller than the entire antibody, such as a Fab lacking the Fc region, preserves the aptamer’s ability to deliver the antibody cargo through the BBB. In such a case the construct could exert therapeutic benefit on brain tumors or brain metastases of breast cancer, while avoiding the side effects previously seen with antibodies entering the brain [79], thanks to the lower dose of the antibody dispensed in a conjugated form.

It is still debated whether BRCA1- and BRCA2-deficiency, which causes genomic instability and increased tumor burden, could increase immunosensitivity in breast cancer and predict clinical benefit from immunotherapy [80, 81]. In the present study, we used two BRCA1- and BRCA2-proficient cell lines [82, 83]. Upcoming studies with a larger number of TNBC cell lines, either wild-type or mutated for the BRCA genes, will be helpful to answer whether BRCA1- and BRCA2-mutations might influence our combined aptamer-immunotherapy.

## Conclusions

PDGFRβ inhibition by a high efficacious nuclease-resistant aptamer favors antitumor immunity and potentiates anti-PD-L1 antibody responses on TNBC growth and lung metastases formation. We first propose that combining PDGFRβ blockade with anti-PD-L1 immune checkpoint inhibitors may be a viable therapeutic approach for triple-negative breast cancer.

## Supplementary information

**Additional file 1:** **Figure S1. PDGFRβ Gint4.T aptamer specifically binds to human and mouse lymphocytes.** Cell-ELISA assays on activated human (**A**) and mouse (**B**) lymphocytes with 5'-biotinylated PDGFRβ Gint4.T aptamer at the indicated concentrations. The background binding value for 5'-biotinylated Scr aptamer was subtracted from each data point. Data shown are mean ± SD (*n*=3). **Figure S2. Similarity of human and mouse PDGFRβ**. (**A**) The dotplot matrix shows the sequence similarity between human and mouse PDGFRβ protein obtained by using Dotmatcher tool (EMBOSS) with a scoring matrix Blosum 62 and a threshold line of 13. (**B**) NIH3T3 cells were mock-treated or serum-starved (ss) and then left untreated (-) or stimulated with PDGF-BB, as indicated. Cell lysates were immunoblotted with the indicated antibodies. Molecular weights of indicated proteins are reported. **Figure S3. PDGFRβ and PD-L1 immunohistochemical expression**. Shown are images from one representative tumor for each treatment group immunostained with anti-PDGFRβ (**A**) and anti-PDL1 (**B**) antibodies. Magnification 40 ×, scale bar = 50 μm.

## Data Availability

All data generated or analyzed during in this study are included in this published article.

## Abbreviations

2’F-Py: 2’Fluoro-pyrimidines
ATCC: American Type Culture Collection
DAPI: 4′,6-Diamidino- 2-phenylindole
BB: Binding buffer
BBB: Blood-brain barrier
DPBS: Dulbecco’s phosphate-buffered saline
BM-MSCs: Bone marrow-derived mesenchymal stem cells
HER2: Epidermal growth factor receptor 2
EMT: Epithelial-mesenchymal transition
ERK1/2: Extracellular-signal regulated kinase 1/2
FBS: Fetal bovine serum
GBM: Glioblastoma
H&E: Hematoxylin and eosin
hPBMCs: Human peripheral blood mononuclear cells
IFN-γ: Interferon gamma
IL-2: Interleukin-2
LDH: Lactate dehydrogenase
mAbs: Monoclonal antibodies
MSCs: Mesenchymal stem cells
PDGFRβ: Platelet-derived growth factor receptor β
PD-L1: Programmed cell death-ligand 1
PI3K: Phosphatidyl-3-kinase
RPMI-1640: Roswell Park Memorial Institute-1640 medium
RT: Room temperature
RTKs: Receptor tyrosine kinases
RT-qPCR: Reverse transcription quantitative polymerase chain reaction
TILs: Tumor-infiltrating lymphocytes
TME: Tumor microenvironment
TNBC: Triple-negative breast cancer
