# Supplementary material for: Aptamer targeted therapy potentiates immune checkpoint blockade in triple-negative breast cancer
Source: J Exp Clin Cancer Res. 2020 Sep 7;39:180. doi: 10.1186/s13046-020-01694-9 (PMC7487859; doi:10.1186/s13046-020-01694-9)
Supplement: Supplementary file 1 — Additional file 1: Figure S1. PDGFRβ Gint4.T aptamer specifically binds to human and mouse lymphocytes. Cell-ELISA assays on activated human (A) and mouse (B) lymphocytes with 5'-biotinylated PDGFRβ Gint4.T aptamer at the indicated concentrations. The background binding value for 5'-biotinylated Scr aptamer was subtracted from each data point. Data shown are mean ± SD (n=3). Figure S2. Similarity of human and mouse PDGFRβ. (A) The dotplot matrix shows the sequence similarity between human and mouse PDGFRβ protein obtained by using Dotmatcher tool (EMBOSS) with a scoring matrix Blosum 62 and a threshold line of 13. (B) NIH3T3 cells were mock-treated or serum-starved (ss) and then left untreated (-) or stimulated with PDGF-BB, as indicated. Cell lysates were immunoblotted with the indicated antibodies. Molecular weights of indicated proteins are reported. Figure S3. PDGFRβ and PD-L1 immunohistochemical expression. Shown are images from one representative tumor for each treatment group immunostained with anti-PDGFRβ (A) and anti-PDL1 (B) antibodies. Magnification 40 ×, scale bar = 50 μm. [file 13046_2020_1694_MOESM1_ESM.pdf]

Supplementary Information

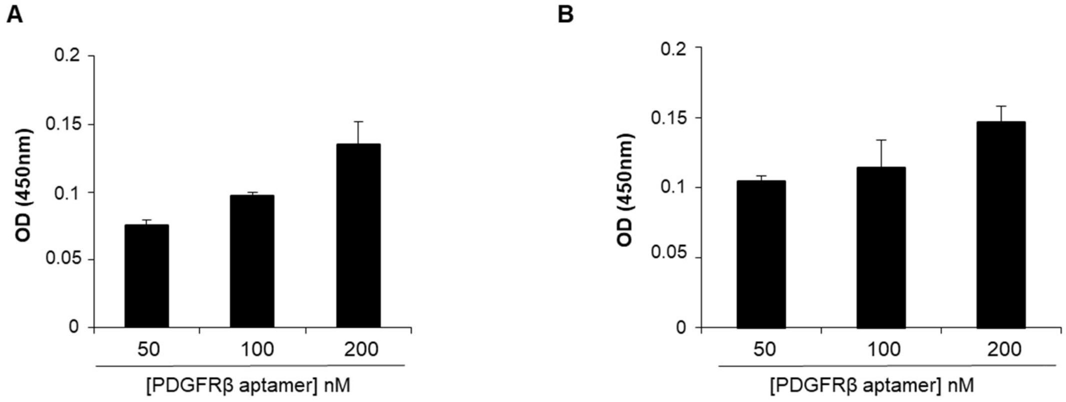

Figure S1. PDGFRβ Gint4.T aptamer specifically binds to human and mouse lymphocytes. Cell-ELISA assays on activated human (**A**) and mouse (**B**) lymphocytes with 5'-biotinylated PDGFRβ Gint4.T aptamer at the indicated concentrations. The background binding value for 5'-biotinylated Scr aptamer was subtracted from each data point. Data shown are mean ± SD (n=3).

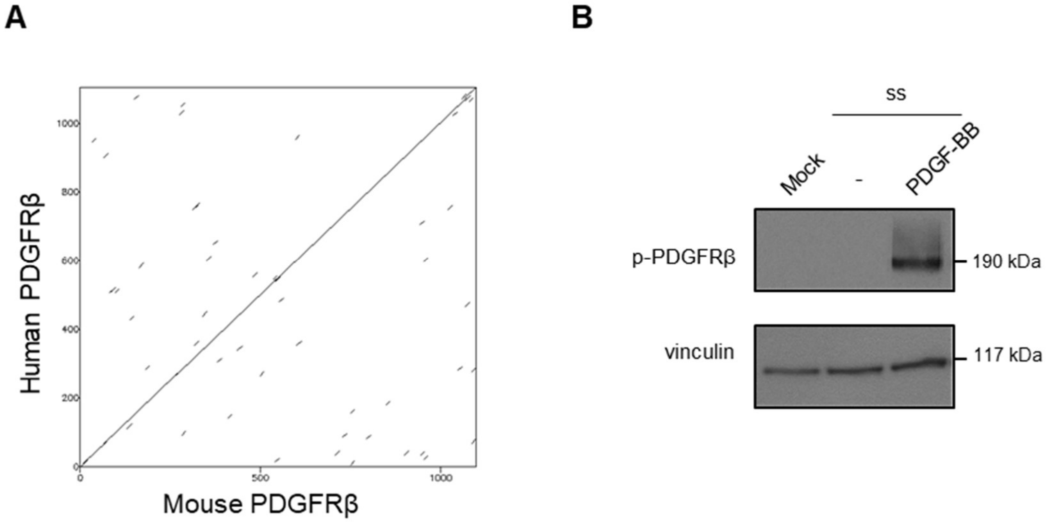

Figure S2. Similarity of human and mouse PDGFRβ. (**A**) The dotplot matrix shows the sequence similarity between human and mouse PDGFRβ protein obtained by using Dotmatcher tool (EMBOSS) with a scoring matrix Blosum 62 and a threshold line of 13. (**B**) NIH3T3 cells were mock-treated or serum-starved (ss) and then left untreated (-) or stimulated with PDGF-BB, as indicated. Cell lysates were immunoblotted with the indicated antibodies. Molecular weights of indicated proteins are reported.

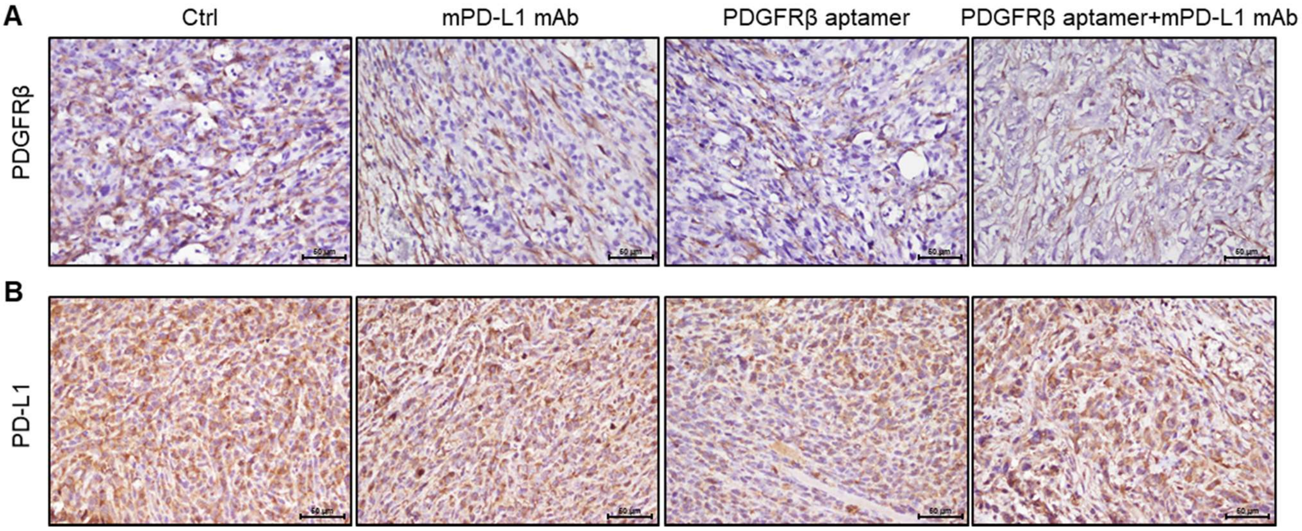

Figure S3. PDGFRβ and PD-L1 immunohistochemical expression. Shown are images from one representative tumor for each treatment group immunostained with anti-PDGFRβ (**A**) and anti-PD-L1 (**B**) antibodies. Magnification 40 ×, scale bar = 50 μm.
